# Screening for subclinical genital herpes in pregnant females - A neglected practice

**DOI:** 10.12669/pjms.41.2.10045

**Published:** 2025-02

**Authors:** Kainat Farrukh, Saima Zaki, Farhan Rasheed, Sumaira Niaz, Maham Javed, Nadia Naseem

**Affiliations:** 1 Kainat Farrukh Department of Histopathology, University of Health Sciences, Lahore, Pakistan; 2 Saima Zaki Department of Obstetrics and Gynecology, Jinnah Hospital Lahore, Pakistan; 3 Farhan Rasheed Department of Microbiology, Allama Iqbal Medical College, Lahore, Pakistan; 4 Sumaira Niaz Department of Histopathology, University of Health Sciences, Lahore, Pakistan; 5 Maham Javed Department of Histopathology, University of Health Sciences, Lahore, Pakistan; 6 Nadia Naseem Department of Histopathology, University of Health Sciences, Lahore, Pakistan

**Keywords:** Cell block, Herpes simplex virus, Immunofluorescence, Immunocytochemistry, Immunohistochemistry, Liquid-based cytology, Pregnant females

## Abstract

**Objective:**

The present study was aimed to screen the Herpes simplex virus (HSV) infection in cervical smears of clinically suspicious to asymptomatic pregnant women of local population.

**Method:**

This descriptive cross-sectional study was conducted at the Department of Histopathology, University of Health Sciences Lahore, Pakistan from August 2021 to September 2022. A total of N=120 cervical smears were taken from the pregnant females of gestation age 18-38 weeks, visiting the outpatient department (OPD) of Gynecology & Obstetrics, Jinnah Hospital Lahore. Endocervical smears were taken; cell block and cytospin preparations were prepared. The cytological changes were categorized according to the updated Bethesda Classification System 2014 and the samples were screened for the presence of HSV 1 & 2 through immunocytochemistry (ICC), immunohistochemistry (IHC) and Immunofluorescence (IF). SPSS version 25.0 was used to analyze the data and p-Value of ≤ 0.05 was considered as statistically significant.

**Results:**

Out of N=120 pregnant females, 12.5% were positive for HSV on ICC while 2.5% cases showed positive nuclear staining for HSV on cell block by IHC. On IF staining, around 7.5% samples were positive for HSV by cytospin method whereas 15% cases showed positivity for HSV on cell block method. Typical herpetic genital lesions were observed in 13% of HSV positive females (p=0.043). Pap staining of cervical smears revealed negative for intraepithelial lesion or malignancy (NILM) in 90% of the samples while 10% of the cases were suggestive of atypical squamous cells of undetermined significance (ASCUS). Moreover, 13% cases showed candida albicans on culture test. None of the subjects showed positive Trichomonas vaginalis on wet smears.

**Conclusion:**

Early and effective diagnosis of sub-clinical cervico-vaginal HSV infection in pregnant females by adopting minimally invasive cytological techniques and immunofluorescent staining may lead to reduced morbidity and mortality.

## INTRODUCTION

According to World Health Organization (WHO) reports, globally around 3.7 billion people under age 50 (67%) suffers from herpes simplex virus Type-1 (HSV-1) infection, which is the key cause of oral herpes whereas approximately 491 million individuals aged 15-49 (13%) have herpes simplex virus Type-2 (HSV-2) infection universally which is the foremost source of genital herpes.^1^ Herpes infection may develop primary infection or latency, and cause oral and genital herpes manifesting as conjunctivitis, eczema herpeticum, and other diseases. Primary infection in the mother causes disseminated herpes more commonly in preterm infants.^2^ Primary genital herpes simplex virus (HSV) acquired to pregnant females are at more high risk of transferring neonatal herpes to infants as they are continuously shedding viruses, associated with women who have recurrent genital herpes.^3^

HSV-2 infection surges the risk of having HIV infection by almost three-fold. Furthermore, people suffering from both HIV and HSV-2 infection are more expected to spread HIV to others. Pregnant women, when acquire primary HSV infections, are more prone to severe manifestations as compared to non-pregnant ones. Specifically, gingiva-stomatitis and vulvo-vaginitis herpetica are likely to disseminate with visceral involvement such as hepatitis, encephalitis, thrombocytopenia, leucopenia and coagulopathy especially during the third trimester with a 50% mortality rate.^4^ Females with initial genital herpes can shed the infection without symptoms. Genital HSV shedding in asymptomatic HSV-2 infected people occur within 10.2% of days, as compared to 20.1% of days in symptomatic infected people.^3^

### Cervical cytology:

It plays an important role in the diagnosis of cervical infections. Pap smear test is the most cost-efficient method for the prevention and diagnosis of cervical cancer. But with advancement in methodology, it has been seen that the pap smears has shown high false-negative rates due to sampling errors, presence of obscuring materials, screening and interpretation errors.^5^ Liquid-based cytology (LBC) was presented in mid-1990s as another method to process cervical samples.^6^ However, there are certain limitations of LBC, most important of which is high cost and trained cytotechnicians. Manual Liquid Based Cytology (MLBC) is a procedure that suspends cells in a single layer, thus increases the detection of precursor lesions and has lower false-negative rate therefore, improves the specimen adequacy.^7^ Recently, a broadly applied method used is the direct fluorescent antibody assay (DFA) for direct detection of HSV in clinical specimens. The variability of performance of assay depends on quality of sample and competences of the testing personnel. The asset of the assay includes brief testing time (hours) and comparatively lower cost.^8^ The cytological findings of HSV 1 and HSV 2 infections are generally similar and cannot reliably differentiate between the two types based on cytology alone. Both types of HSV produce characteristic cytopathic effects in infected epithelial cells, which are identified in cytological smears. However, the distinction is typically made through molecular or serological tests rather than cytological findings. Hence, for HSV detection, serological or other sensitive techniques can be engaged for definite diagnosis.^9^,^10^

HSV infection is one of the most common sub-clinically transmitted infections and its dissemination is a frequent cause of mortality in Pakistan. Unfortunately, no antenatal screening option for HSV is available in any tertiary care hospital of Pakistan. Moreover, there is no baseline serological data for HSV infection during pregnancy in our population. This study was therefore designed to determine the cytological changes in pregnant females according to the latest Bethesda Classification 2014 and to detect genital HSV 1 & 2 (clinical or subclinical) through direct immunofluorescence and immunohistochemistry on cervical smears and cell blocks.

## METHODS

A descriptive cross-sectional study was conducted from August 2021 to September 2022 consisting of total of N=120 pregnant females with of child-bearing age (18-45 years) presenting at the antenatal clinic of Jinnah Hospital, Lahore after taking written informed consent. Convenient sampling technique was used to collect the samples.

### Inclusion & Exclusion Criteria:

For pap smear testing, gravida pregnant females of ≥18-34 weeks were selected whereas pregnant females with complicated or precious pregnancy, chronic debilitating illness like uncontrolled diabetes, uncontrolled hypertension, tuberculosis, bleeding disorders, chronic immune disorders like systemic lupus erythematosus, rheumatoid arthritis, grave’s disease etc. and unsatisfactory smears were excluded from the study.

### Ethical approval:

This study was approved by Ethical Review Committee of University of Health Sciences Lahore Pakistan (UHS/REG-20/ERC/457; Dated: February 12, 2020). After taking written informed consent from the patients, a detailed history was taken using a predetermined proforma that included main complaint, demographic details and obstetrics profile of the patient. For Pap smear sampling, the subjects were placed in lithotomy position and a sterile cervical swab was taken by Cytobrush (SurePath®) from endocervical and periurethral area by an experienced gynecologist and submitted in transport medium for culture sensitivity.

At least three endocervical samples of each participant were taken through brush cytology. One brush sample was smeared on one half of three labeled slides which were placed in fixative solution for 30 seconds followed by air-drying. The detachable head of Cytobrush was dropped in labelled SurePath^®^ vial for LBC. Third brush sample was spread on glass slide to see Trichomonas vaginalis on a microscope wet preparation at the bed side. At 400x magnification, the morphology of trichomonas can be clearly seen and is identified by its characteristic flagellation.^11^

The prepared slides were labelled and transported to Department of Histopathology, University of Health Sciences Lahore for further processing. After taking the sample on brush, sedimentation process was performed to eradicate debris, followed by centrifugation to form a pellet, a fraction of which was later spread over to the slide in a monolayer intended for examination. The slides were then stained with Papanicolau stain for classification of changes according to the updated Bethesda system 2014.^12^

### Cell blocks preparation:

Cell blocks were prepared by Plasma thrombin method.^13^ The pellet prepared from centrifuged cell suspension was enmeshed into a clot with all the cellular material by adding equal proportions of thrombin and plasma. The clot was placed into a labeled cassette for histopathological examination.

### HSV-1 and HSV-2 Immunofluorescence:

Light Diagnostic SimulFluor HSV1/2 (DFA KIT, #3293) was used as per manufacturer’s instructions. HSV-1 infected cells revealed apple-green fluorescence and HSV-2 infected cells revealed yellow gold fluorescence. The uninfected cells stained dull red owing to the presence of Evans blue in the SimulFluor HSV1/2 reagent.

### Immunocytochemistry and Immunohistochemistry for HSV 1&2:

Immunohistochemistry and immunocytochemistry were carried out using antibodies for HSV-1 (Rabbit polyclonal Ab#084P; BioGenex, San Ramon CA) and HSV-2 (Rabbit polyclonal Ab#085P; BioGenex) on smears and sections (from cell block preparations) following the manufacturer’s protocol. Strong diffuse nuclear and cytoplasmic staining of infected cells having HSV1, or HSV2 was considered as positive.^14^

### Statistical analysis:

The data was entered and analyzed using SPSS 25.0. Mean +SD were given for quantitative variables. Frequencies and percentages were given for qualitative variables. Chi-square test was applied between qualitative variables; Fisher exact test was applied between qualitative and quantitative variables. A p-value of ≤0.05 was considered as statistically significant.

## RESULTS

In this study, age group of subjects ranged from 18 - 45 years with a mean age of 26.4 ± 5.1 years. An increased trend in age with seroprevalence of HSV 1&2 was observed in the data although it did not show any significant association (p-Value=0.184). All the pregnant females presented with complain of discharge. According to the history of color of discharge, majority of the females (62.5%; n=75) complained of whitish discharge while 2.5% (n=3) reported infected greenish discharge. Around n=75 (62.5%) subjects had noticed the discharge for the first time in pregnancy while 42 (35%) females reported similar discharge in their previous pregnancies as well. Out of 120 cases, 18 (15%) were positive for HSV-1 and 2 where 3/18 females had typical genital lesions on clinical examination findings. The presence of genital lesions in females showed significant association (p= 0.043) with HSV-1 infectivity (Table-I). Fifty-two percent of pregnant females (n=63) presented with gingivitis of which 20% (n=24) complained of erythematous/ bleeding gums, 17.5% (n=21) with friable gums and 15% (n=18) with swollen gums.

**Table-I:** Association of Genital lesions with HSV 1 &2 infectivity.

Risk factors	Variables	Frequency [N (%)]	Test applied	P-Value
Age	Pregnant females of age 18 - 45 years	120	Chi-square test and Fisher exact test	0.184
Genital lesions	Yes	15 (13%)		0.043*
	No	105 (87.5%)		
Abortion	Yes	63 (52.5%)		0.845
	No	57 (47.5%)		
History of cold sores	Yes (stage 1)	72 (60%)		0.236
	Vesicular lesions	10 (12%)		
Obstetric history	Conceived <4 times	99 (82.5%)		0.142
	Conceived >4 times	21 (17.5%)		

* p-Value of ≤ 0.05 was considered as significant.

**Fig.1:**
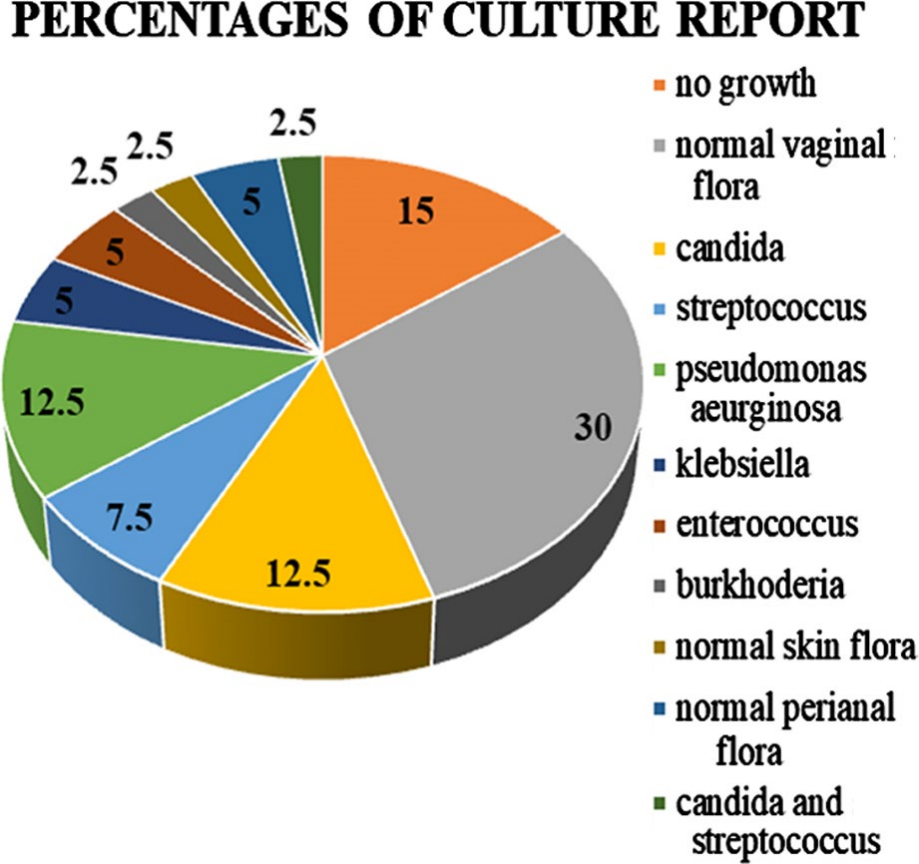
Pattern of microbiological pathogens on culture sensitivity (data as shown as percentages).

The culture report of majority of females (30%; n= 36) showed normal vaginal flora. In addition, candida *albicans* was reported in 12.5% (n=15) females who had whitish smelly discharge. Culture from the females with symptomatic urinary tract infections revealed positive Pseudomonas *aeruginosa* and Klebsiella organisms in 12.5% (n=15) and 5% (n=06) cases (Fig.1). None of the subjects showed positive Trichomonas *vaginalis* on wet smears.

**Fig.2:**
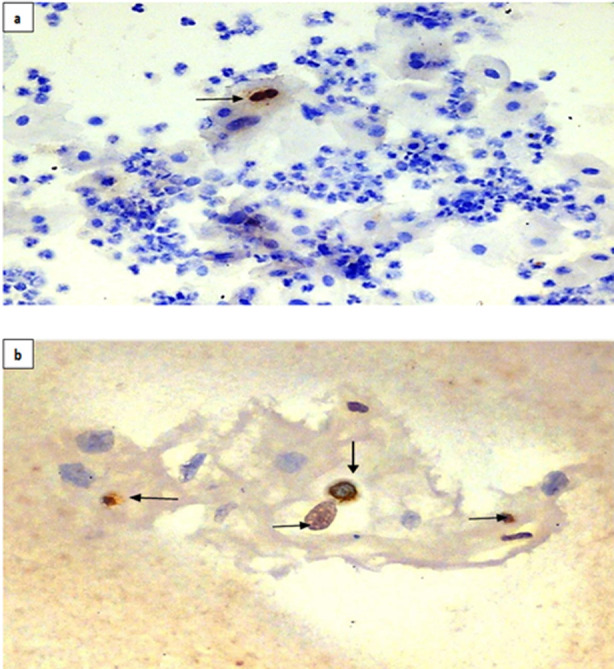
a) Photomicrograph showing squamous cell with binucleation (arrow) and positively stained nuclei for HSV1/2 (ICC; 20X); b) Photomicrograph showing of infected HSV1/2 positive binucleate and mononucleate cells (arrow) (IHC; 20X).

Cytological findings on conventional pap smears in majority (n=108; 90%) revealed NILM, while morphology suggestive of ASCUS was seen in n=12 (10%) cases. Smears with moderate to severe inflammation comprising of neutrophils and lymphocytes in background were also noted. Presence of normal vaginal flora, Lactobacilli in background of smears were included in NILM. Whereas cytological changes evocative of LSIL, comprising nuclei 2.5-3 times the size of normal intermediate cell nucleus with or without minimal nuclear hyperchromasia and mildly asymmetrical nuclear contours or poorly shaped cytoplasmic halos or vacuoles, similar to koilocytes, presence of nucleoli and increased mitotic activity were categorized as ASCUS. On ICC, 15 cases (12.5%) were positive for HSV while three cases (2.5%) showed positive nuclear staining for HSV1/2 on cell block by IHC (Fig.2 a & b). These smears and sections showed strong positive nuclear staining with the antibody.

**Fig.3:**
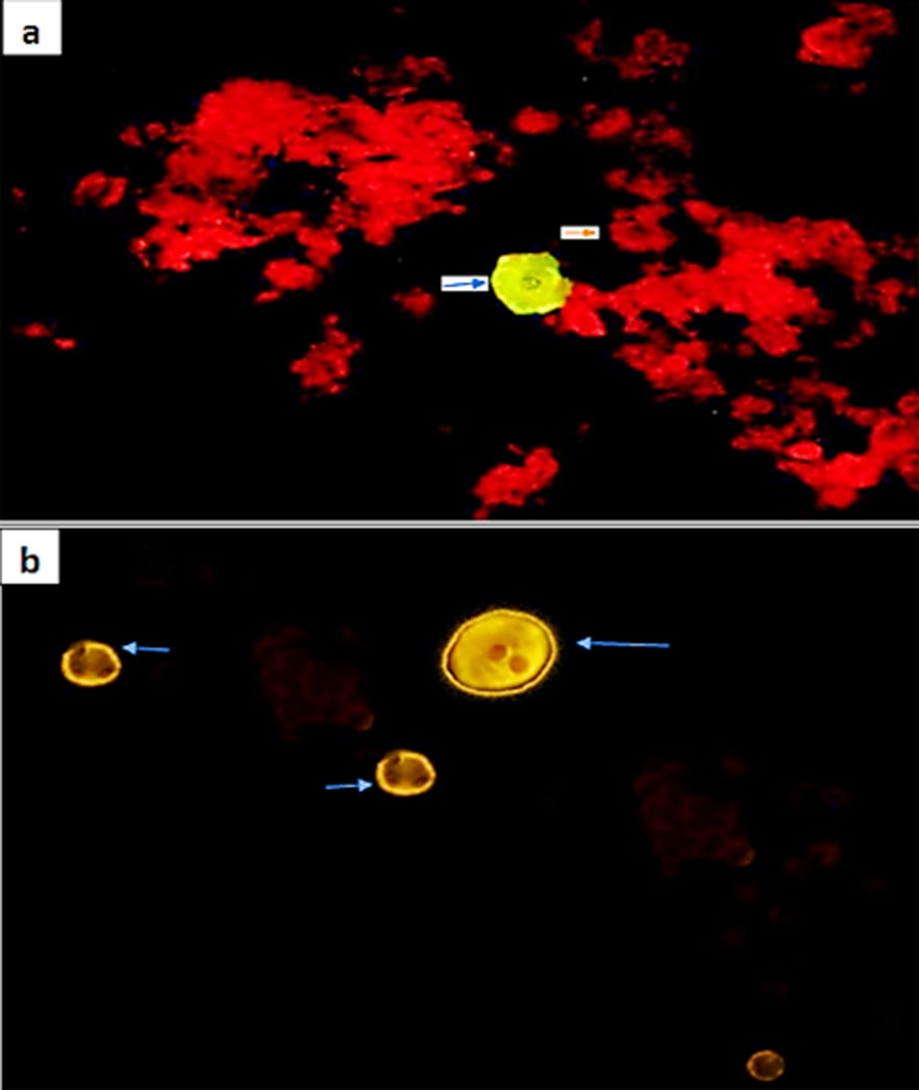
Photomicrograph showing positive HSV-1 squamous cell, apple green fluorescence (blue arrow) and uninfected cell as dull red (orange arrows) in background on cell block (IF; 20X); b) Photomicrograph showing HSV-2 infected cells (blue arrow) with yellow-golden fluorescence on cytospin (IF; 40X).

The SimulFluor DFA kit has monoclonal antibodies for both HSV-1 and 2 which attaches to 155KD major capsid protein in HSV infected cells. FITC (Fluorescein isothiocyanate) filter was used which give apple-green fluorescence for HSV-1 infected cells and yellow-gold fluorescence for HSV-2 infected cells. The uninfected cells stained dull red. On IF by cytospin method, a total of nine cases (7.5%) were HSV positive. Out of these, six cases (66.6%) were positive for HSV1 while three cases (33.3%) showed positive nuclear staining for HSV2. Whereas on IF by cell block method, a total of 18 cases (15%) were positive for HSV1 and HSV2. Out of these 18 cases, 12 (66.6%) were positive for HSV1 and 6 (33.3%) were positive for HSV2 (Fig.3 a & b).

## DISCUSSION

In the present research, the mean age of the pregnant females diagnosed with HSV infection was 30.8 ± 5.07 years and an increasing trend in age with seroprevalence of HSV 1 &2 was observed in the data although it did not show any significant association (p value=0.184). A study was conducted on a population residing in Iraq, Jordan, Lebanon, Palestine, and Syria and Qatar, which stated HSV-1 seroprevalence increased steadily with age.^15^ The frequency of abnormal vaginal discharge was 67.5% (n=27) in this study. In concordance with the present study, Shah et al.^16^ reported a much higher frequency (78.5%) of pregnant females with abnormal vaginal discharge on clinical examination. On culture, candida infection was reported in 12.5% females however none of the patient was reported positive for trichomonas.

The frequency of fungal infection in the current study is two times higher than the one reported by a local study (6.5%) by Bukhari et al.^17^ In the current study, n=15 (13%) pregnant females presented with the history of typical herpetic genital lesions that demonstrated a significant association (p= 0.043) with HSV positivity. On the contrary, a study conducted in Turkey reported an extremely higher (73.8%) seroprevalence of HSV in pregnant women with genital lesions.^18^ All subjects in the current study either presented with one of the symptoms or were asymptomatic (n=104). The frequency of HSV positivity is lower in the present study as compared to another regional study conducted in Khyber Pakhtunkhwa, Pakistan where HSV was detected in 46.9% of pregnant females by ELISA.^19^

In the current study, only six cases were reported as ASCUS by pap smears whereas LBC reported nearly double (n=12, 10%) cases as ASCUS which is in concordance with the literature that supports better rate of detection of epithelial cell abnormalities using LBC method. A study published by Santwani et al.^20^ reported that by using cell block method, the diagnostic yield for malignancy was raised by 10% when compared to the conventional method in peritoneal washing samples.

The findings of the present study however showed conflicting results with two techniques; only three sample s (2.5%) showed presence of HSV 1&2 antigen on IHC by cell block method while 15 samples (12.5%) were positive for HSV 1&2 on ICC. The decreased sensitivity of cell block in the present study may be referred to the plasma thrombin method hampering the antigenicity of the viral proteins. Furthermore, fixative like formalin is not used but saline is used in the preparation of plasma-thrombin based CBs thus, the cellular yield, cytomorphologic detail, or architectural features are conceded as cell lysis is amplified with saline, which is not a perfectly isotonic cell medium. A local study from Peshawar reported a much lower frequency (1.1%) of HSV-IgM antibodies on serology in pregnant females.^21^ According to various studies conducted in India, the prevalence of HSV antibodies ranges from 5.8% to 65% in pregnant females.^22^,^23^

Another diagnostic test used in the present study is Immunofluorescence (IF) that was also carried out on the specimens obtained from two cytological techniques: cytospin and cell block. The IF technique differentiated HSV-1 and HSV-2 infected cells by exhibiting different fluorescent colors. IF is extensively used for the rapid diagnosis of viral infections by recognition of virus antigen in clinical specimens and recognition of virus-specific antibodies.^24^ In the present study, n=9 samples (7.5%) were positive for HSV on cytospin, and n=18 samples (15%) were positive on cell block technique.

The frequency of HSV-1 in pregnant females was 66.6% while HSV-2 was positive in 33% females. Recently, the epidemiology of HSV is in transition and literature shows that the genital and neonatal herpes is caused not only by HSV-2 but HSV-1 positive cases are also on rise.^25^ It is reported that the incidence of genital HSV-1 is on rise in women of reproductive age.^26^ The present study also highlighted the increased tendency of HSV-1 positivity in genital herpes in pregnant females. Results of an Italian study also reported higher prevalence (91.2%) of HSV-1 in serum of pregnant females by ELISA.^27^ The prevalence of HSV-2 was reported to be 31.4% in pregnant females of Haiti which is quite comparable to the findings of the present study.^28^

The findings of this study could serve as a foundation for public health education campaigns, raising awareness about HSV in pregnancy and improving health literacy among healthcare providers and the community. A critical yet often overlooked aspect of maternal health was investigated by emphasizing on subclinical genital herpes in pregnant females. Moreover, the study’s main focus on both symptomatic and asymptomatic women ensured a comprehensive understanding of the prevalence and presentation of Herpes simplex virus (HSV) infections and its association with different clinico-pathological parameters. The study has promoted a preventative approach to healthcare by highlighting the importance of screening, which can lead to early diagnosis and treatment of sub-clinical cervico-vaginal infections which in turn can reduce morbidity and neonatal transmission risks, significantly improving maternal and neonatal health outcomes. It has also highlighted the need for integrating advanced diagnostic techniques and routine cervical screening into antenatal care, thereby adopting multidisciplinary healthcare practices.

### Limitations:

Due to cost-related issues of SurePath, repeated sampling by using trouble-shooting techniques couldn’t be performed. Moreover, the study’s follow-up was hindered due to non-cooperation of the patients throughout the study.

## CONCLUSION

Herpes simplex virus trends as a sub-clinical infection in pregnant females of our population. Lower immunity and poor socio-economic status of these females calls for emphasizing the screening for HSV1 &2 in all pregnant females to prevent the adverse outcomes during pregnancy and in the neonates.

### Recommendations:

Following recommendations can be made that can reduce morbidity and mortality associated with cervico-vaginal infections in pregnant females***:***

1. Timely cervical screening and health education can significantly reduce the incidence and progression of herpes virus infections.
2. Early and effective diagnosis of cervico-vaginal infections by adopting advanced laboratory techniques, especially sub-clinical HSV infections in pregnant females, is crucial that can reduce the incidence of acquisition and progression of herpes virus.
3. Timely gynecological referral and treatment must be ensured.
4. Preventative educational strategies must be promoted to raise awareness and reduce risks related to sub-clinical HSV infections in pregnant females.

### Authors’ Contributions:

**KF: C**onception and drafting of manuscript, data acquisition and interpretation.

**SZ:** Data acquisition and interpretation; Co-supervision of work.

**FR:** Handling of cytological samples, critical review.

**SN:** Cytological staining, immunohistochemistry and immunofluorescence techniques. Critical analysis.

**MJ:** Drafting of manuscript, data analysis, revising the manuscript critically.

**NN:** Conception of study, revising the manuscript critically, supervision of all the pathological techniques, diagnosis and collaborations, final approval of the version to be published.

All authors are responsible and accountable for the accuracy or integrity of the work.
